# Positive association between blood ethylene oxide levels and metabolic syndrome: NHANES 2013-2020

**DOI:** 10.3389/fendo.2024.1365658

**Published:** 2024-04-18

**Authors:** Chunqi Zhou, Senlin Wang, Lingling Ju, Ruimin Zhang, Yunning Yang, Yanjun Liu

**Affiliations:** ^1^ College of Medicine, Southwest Jiaotong University, Chengdu, Sichuan, China; ^2^ Department of General Surgery, The Third People’s Hospital of Chengdu, Affiliated Hospital of Southwest Jiaotong University, College of Medicine, Southwest Jiaotong University, Chengdu, Sichuan, China

**Keywords:** metabolic syndrome, ethylene oxide, inflammation, epidemiology, NHANES

## Abstract

**Purpose:**

The exposure of Ethylene oxide (EO) is linked to systemic inflammatory response and various cardiovascular risk factors. Hemoglobin’s binding to ethylene oxide (HbEO) was used to measure serum EO level. This research aims to explore the association between metabolic syndrome (MetS) and HbEO, and between HbEO and components of metabolic syndrome.

**Method:**

This research included 1842 participants from 2013 to 2020 in National Health and Nutrition Examination Survey (NHANES) database. Weighted logistic regression models were used to analyze the relationship between HbEO and metabolic syndrome risk, using odds ratio (OR) and 95% confidence interval (CI). The restricted cubic spline plot explores whether there is a dose-response relationship between HbEO and MetS risk. Subgroup analysis was performed to analyze study heterogeneity.

**Results:**

Significant differences were found in gender, educational level, marital status, diabetes status and hypertension among different groups (*P* < 0.001, *P* = 0.007, *P* = 0.003, *P* < 0.001, *P* < 0.001, respectively). The serum HbEO level exhibited positive correlation with metabolic syndrome risk in Q2 level (OR=1.64, 1.04~2.48), Q3 level (OR=1.99, 1.29~3.08), and Q4 level (OR=2.89, 1.92~4.34). The dose-response association suggested a possible linear association between serum HbEO and metabolic syndrome risk (*P*-overall=0.0359, *P*-non-linear=0.179). L-shaped association was found between HbEO and the risk of MetS in female population, obese population and mid-age and elder population (*P*-overall<0.001, *P*-non-linear=0.0024; *P*-overall=0.0107, *P*-non-linear=0.0055 *P*-overall<0.001 *P*-non-linear=0.0157).

**Conclusion:**

This study indicates a linear correlation between MetS and HbEO, with MetS risk escalating as HbEO levels increase. The prevalence of MetS varies depending on BMI, age and gender, and these factors can also influence MetS prevalence when exposed to EO.

## 1 Introduction

Metabolic syndrome (MetS) is defined as a pathologic condition characterized by abdominal obesity, insulin resistance, hypertension, and hyperlipidemia (1). MetS is a critical health issue that elevates the likelihood of individuals developing heart disease, diabetes, stroke, and conditions linked to the buildup of fatty deposits in the walls of arteries, known as atherosclerosis (2–4). The prevalence of MetS has been studied in various countries. In the US, Zimmet et al. (5) found that it increased from 32.5% in 2011 to 36.9% in 2016, while Hirode et al. reported a prevalence of 34.7%. In European countries, the prevalence varied from 12% to 26% (6). In China, the prevalence increased from 8% in 1992 to 10.6% in 2002 in urban areas and from 4.9% in 1992 to 5.3% in 2002 in rural areas. The prevalence of MetS in China was estimated to have increased to 15.5% in 2017 (7). Currently, there is no global data available on MetS. However, its prevalence is approximately three times higher than that of diabetes, therefore, the global prevalence of MetS is estimated to be a quarter, and for adults over 40 years old, the prevalence is around 40% (8, 9). Furthermore, the prevalence of MetS is correlated with the prevalence of obesity. As obesity becomes increasingly common, MetS has emerged as a significant public health concern (6, 8).

Ethylene oxide (EO) is present throughout in the environment, deriving from sterilized medical equipment, fumigated food, cosmetics, and inhalation of contaminated air, tobacco smoke and car exhaust fumes (10, 11). EO is a direct-acting alkylating agent, and acute exposure to EO can cause nausea, bronchitis, and pulmonary edema; Chronic long-term exposure increases the risk of neurological disorders and cancer (12, 13). EO can induce dose-related increases in hemoglobin adduct frequencies, genetic mutations, and genetic translocations in exposed rodent germ cells (14, 15). Cytogenetic studies *in vitro* and *in vivo* have confirmed the genotoxicity and mutagenicity of EO. Studies have also provided substantial evidence of carcinogenicity to rodents (16). Exposure to EO is linked to systemic inflammatory response (17, 18) and various cardiovascular risk factors such as smokers, serum lipid levels and diabetes (3, 16, 19–21). With the widespread industrialization and extensive use of chemical substances, there is an increasing interest in understanding the relationship between environmental factors such as EO and kinds of MetS. Xu Zhu et al. (22) found hemoglobin's binding to ethylene oxide (HbEO) was positively associate with total cholesterol (TC), total triglycerides, low-density lipoprotein and inflammatory biomarkers but negatively associated with high-density lipoprotein. Jingyu Guo et al. (3) found that higher HbEO levels were significantly associated with an increased prevalence of diabetes mellitus. The group led by Ningtao Wu (23) discovered that HbEO levels are strongly and non-linearly correlated with diastolic blood pressure (DBP). Iokfai Cheang et al. (24) reported that elevated quartiles of HbEO were inversely associated with BMI, WC and obesity following full adjustment.

Since the diseases associated with MetS are the leading causes of morbidity and mortality, identifying the underlying cause of MetS has been the focus of many studies (25). However, a comprehensive understanding of the exact relationship between the MetS and HbEO remains lacking. As EO was proved to be a risk factors for MetS components, it is important to figure out if EO is also a risk factor for MetS. Therefore, this study examines the potential connection between EO exposure and MetS by analyzing data extracted from National Health and Nutrition Examination Survey (NHANES), to unveil plausible pathogenic mechanisms. The insights gained from this research can contribute to a better comprehension of the intricate relationship between chemical substances in modern life and chronic diseases.

## 2 Patients and methods

### 2.1 Study population

**Figure 1:**
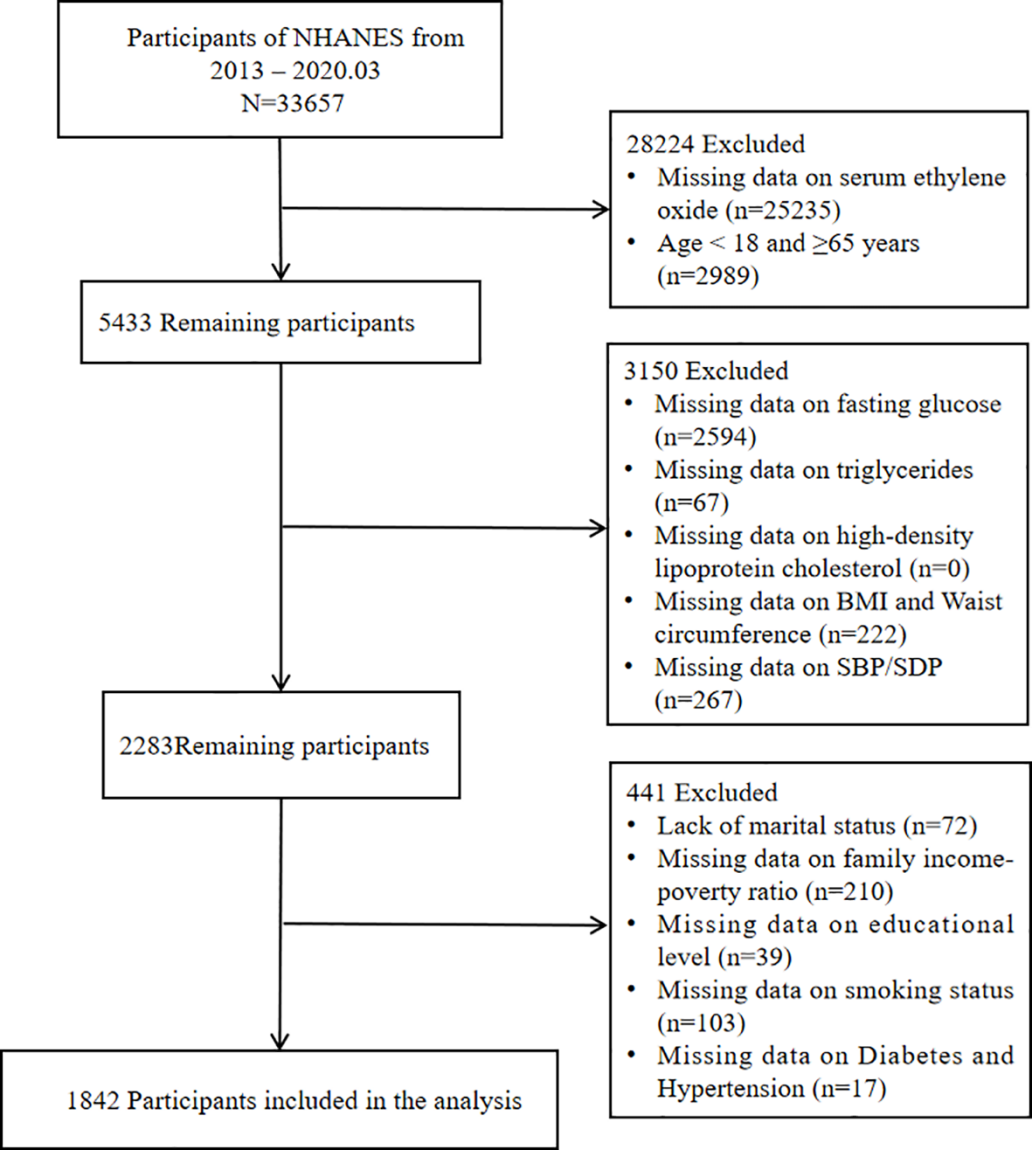
Flow chart of the patients included in the study.

National Health and Nutrition Examination Survey (NHANES) is an ongoing, nationally representative series of surveys conducted every two years to monitor the health and nutritional status of non-institutionalized citizens in the United States (26). The study cohort was confined to the survey period from 2013 to March 2020, as EO measurements were unavailable before this timeframe (27, 28). In NHANES 2013-2020, a total of 33657 participants completed both interviews and medical examinations. We selected adults aged 18 to 65 years for the study. According to the NCEP ATP III-2005 criteria, the components defining metabolic syndrome primarily consist of plasma glucose, triglycerides, high-density lipoprotein cholesterol (HDL-C), and waist circumference. Therefore, participants with missing data on the components defining MetS will be excluded. Participants with missing demographic information such as marital status, severe drinking habits, family income-poverty ratio (PIR), educational level, and smoking status were also excluded. Ultimately, our study included 1842 participants (**Figure 1**).

### 2.2 Assessment of ethylene oxide and metabolic syndrome

The modified Edman reaction measures HbEO in human whole blood or red blood cells. This method is applied to N-terminal hemoglobin adducts and has been optimized to enhance product yield, sensitivity, and automation (29). Thus, NHANES staff utilize this method to detect HbEO.

MetS was characterized based on the NCEP ATP III-2005 criteria (30, 31), which entail the presence of three or more of the following conditions: 1) increased waist circumference (EWC), defined as waist circumference ≥102 cm in men and ≥88 cm in women; 2) high blood pressure, indicated by blood pressure levels ≥130/85 mm Hg or the use of medication for previously diagnosed hypertension; 3) reduced levels of HDL-C, with values below <40 mg/dL in men and <50 mg/dL in women, or the use of specific treatment for low HDL-C; 4) elevated triglycerides (TGs), defined as TG levels ≥150 mg/dL or the use of medication for high TG levels; and 5) increased fasting glucose, represented by fasting glucose levels ≥100 mg/L or the use of medication for high glucose levels and a previous diagnosis of type 2 diabetes.

### 2.3 Assessment of covariates

Based on previous research, smoking, and drinking status was categorized into three groups: “never,” “former,” and “current” (32, 33). Hypertension was defined as systolic blood pressure ≥140 mmHg, diastolic blood pressure ≥ 90 mmHg, self-reported physician diagnosis, or current use of antihypertensive medication (34). Information regarding marital status, educational attainment, and ethnicity was extracted from the fundamental demographic data in the NHANES database. Diabetes was delineated by criteria such as glycated hemoglobin (HbA1c) levels equal to or exceeding 6.5%, fasting blood glucose levels greater than or equal to 7 mmol/L, self-reported diabetes, or the present use of antidiabetic medication (35). These subgroups comprise individuals categorized as either normal weight/overweight (BMI < 30 kg/m^2^) or obese (BMI ≥ 30 kg/m^2^) (36). PIR was used to assess household income levels and classified into three groups (<1.3, 1.3 - 3.5, > 3.5) (37). The following data were collected to diagnose MetS: triglycerides, glucose, and HDL-C.

### 2.4 Statistical analysis

To better ascertain the relationship between HbEO levels and MetS, we categorized HbEO levels into four groups using quartiles (Q1<22.62, Q2: 22.62-33.515, Q3: 33.515-148, Q4 >148 pmol/g Hb) (28, 38, 39). Statistical analyses were conducted following the guidelines of NHANES, considering the complex sampling design of the survey to address the bias associated with sample selection, oversampling, and nonresponse (22). Therefore, weights were calculated using the WTSAF2YR weight calculation method for the biochemical markers.

When describing the baseline characteristics of the study population, the data are presented as weighted means ± standard deviation (SD) for continuous measurements, and as unweighted counts along with weighted percentages for categorical measurements. Due to the severe skewness in the distributions of triglycerides and plasma glucose, these variables are presented with the median [interquartile range (IQR)]. The Wilcoxon rank-sum test (ranksum test) was employed to compare independent samples of these variables. Statistical significance was assessed using Student’s *t* test for continuous variables and chi-square tests for categorical variables. Weighted multivariable logistic regression models was used to calculate odds ratios (ORs) with 95% confidence intervals (CIs). The multivariable weighted model was adjusted for age, sex, BMI (non-obese or obese), race, marital status, smoking status, alcohol drinking status, PIR, diabetes, and hypertension. The relationship between HbEO and MetS was also modeled using restricted cubic splines (RCS) with three knots positioned at the 1st, 50th, and 90th percentiles. To assess the robustness of the study, we conducted three sensitivity analyses. Firstly, the International Diabetes Federation-2009 criteria was adopted to redefine the metabolic syndrome (40, 41). Then, we performed the primary analysis on the participants redefined according to these criteria to assess the reliability and robustness of our results. Secondly, due to the skewed distribution of HbEO data, we logarithmically transformed serum ethylene oxide levels (27), and included the transformed log (HbEO) as a continuous variable in two models (38, 39). The study outcomes were defined as MetS according to ATP criteria. Lastly, dietary intake is a significant factor influencing metabolism, and in the United States, dietary quality is determined by the Healthy Eating Index-2015 (HEI-2015) (42). HEI-2015 is used to assess dietary quality based on a population-based scoring algorithm. Evaluation of HEI-2015 comprises 13 dietary components, including Dairy, total protein foods, and seafood and plant proteins, which encompass alternative dairy and protein products (43, 44). We included participants’ total HEI-2015 scores as covariates in the final model to evaluate whether diet quality affects model contributions. It is important to emphasize the use of weighting variable WTDRD1 based on the NHANES analysis guidelines using dietary recall data (excluding missing dietary data n=731, resulting in a final sample size of n=1111) for the third analysis. The visualization of the 13 components was conducted through radar plots (**Supplementary Figure 1**).

To explore potential sources of variability in the relationship between HbEO and the studied outcomes, we extended our investigation through subgroup analyses based on sex, age, non-obese or obese status, hypertension, and diabetes. The selection of specific variables for subgroup analyses was based on their clinical relevance to liver diseases and their potential influence on the relationship between HbEO and the studied outcomes. Including these variables in our analysis allowed us to assess the presence of effect modifications (interactions). This was further examined by incorporating a product term of each stratifying variable and HbEO into the primary model, followed by a Wald test. The entire statistical analysis was conducted using the statistical computing and graphics software R (version 4.1.3) and STATA (version 17.0), with statistical significance set at *P* < 0.05.

## 3 Results

### 3.1 Demographic characteristics of participants

The study involved 1842 participants with an average age of 44 (**Table 1**), comprising 900 male participants and 942 female participants. MetS components include reduced high-density lipoprotein cholesterol, elevated triglycerides, elevated plasma glucose, elevated waist circumference, elevated systolic blood pressure and diastolic blood pressure. The study population was subsequently categorized into two groups: one without metabolic syndrome and another with metabolic syndrome. There were significant differences in MetS components between males and females (*P* < 0.001). Significant differences were also found in educational level, marital status, and diabetes status among different groups (*P* = 0.007, *P* = 0.003, *P* < 0.001, respectively). Furthermore, individuals with MetS were significantly more prevalent in the hypertension group compared to those without (*P* < 0.001).

**Table 1:** Characteristics of participants with available data in the NHANES 2013–2020.

Characteristics	Metabolic Syndrome		
	No (N=1234)	Yes (N=608)	*P values
**Age, Mean (SD), years**	40.48 (13.13)	47.52 (11.27)	<0.001
**Sex, male, n %**	630 (51.5)	270 (50.3)	0.739
**HDL-C, Mean (SD), mg/dl**	57.84 (15.65)	44.16 (10.23)	<0.001
**Triglycerides, Median (IQR), mg/dL**	76 (54-105)	147 (101-219)	<0.001
**Plasma Glucose, Median (IQR), mg/dL**	97 (92-103)	108 (102-123)	<0.001
**Waist circumference, Mean (SD), cm**	88.85 (9.67)	112.40 (12.14)	<0.001
**SBP, Mean (SD), mmHg**	117.36 (14.77)	125.31 (15.36)	<0.001
**SDP, Mean (SD), mmHg**	70.21 (10.60)	75.50 (10.87)	<0.001
**BMI, category, n %**			<0.001
Non-obese	917 (74.3)	188 (27.0)	
Obese	317 (25.7)	420 (73.0)	
**Race, n %**			0.218
Mexican American	167 (9.3)	106 (10.9)	
Non-Hispanic Black	266 (11.9)	136 (11.1)	
Non-Hispanic White	440 (62.3)	230 (64.6)	
Other Race	361 (16.5)	136 (13.4)	
**Educational level, n %**			0.007
High School or below	211 (12.8)	150 (17.1)	
High School Grad/GED or Equivalent	288 (22.9)	148 (29.0)	
College graduate or above	348 (33.1)	110 (21.7)	
Some College or AA degree	387 (31.2)	200 (33.2)	
**Maital status, n %**			0.003
Married/Living with Partner	735 (62.2)	373 (67.0)	
Widowed/Divorced/Separated	182 (14.8)	142 (19.5)	
Never married	317 (23.0)	93 (13.5)	
**Drinking status, n %**			0.177
Former	81 (6.6)	62 (8.7)	
Now	913 (79.0)	422 (73.8)	
Never	240 (14.4)	124 (17.5)	
**Smoking status, n %**			0.347
Former	222 (21.3)	111 (21.6)	
Never	660 (53.3)	296 (48.5)	
Now	352 (25.4)	201 (29.9)	
**Diabetes status, n %**			<0.001
No	1027 (85.4)	264 (46.0)	
Pre-diabetes	66 (3.3)	227 (34.0)	
Diabetes mellitus	141 (11.3)	117 (20.0)	
**Hypertension, yes, n %**	1495 (18.6)	1868 (59.5)	<0.001
**PIR, n %**			0.201
<1.3	775 (24.4)	712 (23.4)	
1.3-3.5	1440 (31.3)	1161 (37.2)	
≥3.5	1632 (44.3)	1098 (39.4)	

BMI, body mass index; PIR, family income-poverty ratio; SBP, systolic blood pressure; DBP, diastolic blood pressure; HDL-C, high-density lipoprotein cholesterol; SD, standard deviation; IQR, interquartile range.

Metabolic Syndrome defined as ATP-III.

*For continuous variables, P-values were calculated using weighted Student’s t test, and for categorical variables, P-values were computed using weighted chi-square tests.

### 3.2 Multivariate weighted logistic analysis between HbEO and MetS

In the multivariate weighted logistic analysis, the investigation focused on assessing the correlation between HbEO levels and the risk of MetS and the correlation between HbEO and the risk associated with individual MetS components. (**Table 2**) The serum HbEO level exhibited a positive correlation with metabolic syndrome risk in Q2 level (OR=1.64, 95% CI: 1.04~2.48), Q3 level (OR=1.99, 95% CI: 1.29~3.08), and Q4 level (OR=2.89, 95% CI: 1.92~4.34) in model 1, with a significant *p*-value for trend (*P*=0.01), indicating that with the level of HbEO increased, the MetS risk increased. Regarding elevated waist circumference, a significantly positive association was found in the Q4 level (OR=2.28, 95% CI: 1.04~5.01) with a significant *p*-value for trend (*P*=0.05) in model 1. As for elevated blood pressure, a significantly positive association was found in Q4 level in model 1 (OR=1.64, 95% CI: 1.07~2.52). Regarding reduced high-density lipoprotein cholesterol, a significantly positive association was found in Q2 and Q4 in model 1 with a significant *p*-value for trend (*P*=0.002) and in Q4 in model 2 (OR=3.03, 95% CI: 1.60~5.75) with a significant *p*-value for trend (*P=*0.04). As for elevated total triglycerides, a significantly positive association was found in Q3 and Q4 in model 1 (OR=1.81, 95% CI: 1.15~2.84; OR=2.78, 95% CI: 1.66~4.65, respectively) with a significant *p*-value for trend (*P*=0.04) and in Q3 and Q4 in model 2 (OR=1.72, 95% CI: 1.05~2.81; OR=2.28, 95% CI: 1.19~4.37, respectively). Regarding diabetes, a significant negatively association was found in Q4 in model 1 (OR=0.62, 95%CI: 0.39~0.99) and in Q3 and Q4 in model 2 (OR=0.57, 95%CI: 0.33~0.98; OR=0.40, 95%CI: 0.19~0.84).

**Table 2:** Multivariate weighted logistics model analysis reveals the association between HbEO levels and the risk of Metabolic Syndrome as well as its components.

Characteristics	MetS		EWC		Hypertension		RHDL-C		ETGs		Diabetes	
EO Group	Model 1 OR (95% CI)	Model 2 OR (95% CI)	Model 1 OR (95% CI)	Model 2 OR (95% CI)	Model 1 OR (95% CI)	Model 2 OR (95% CI)	Model 1 OR (95% CI)	Model 2 OR (95% CI)	Model 1 OR (95% CI)	Model 2 OR (95% CI)	Model 1 OR (95% CI)	Model 2 OR (95% CI)
Q1	1.00	1.00	1.00	1.00	1.00	1.00	1.00	1.00	1.00	1.00	1.00	1.00
Q2	**1.64 (1.04,2.58)**	1.50 (0.86,2.61)	2.04 (0.91,4.59)	1.86 (0.83,4.18)	0.47 (0.53,1.43)	0.76 (0.45,1.27)	**1.70 (1.00,2.87)**	1.52 (0.90,2.55)	1.40 (0.88,2.23)	1.27 (0.79,2.03)	0.86 (0.51,1.46)	0.88 (0.52,1.51)
Q3	**1.99 (1.29,3.08)**	**1.91 (1.13,3.22)**	1.46 (0.73,2.95)	1.17 (0.55,2.51)	1.39 (0.89,2.17)	1.15 (0.72,1.84)	1.31 (0.84,2.04)	1.24 (0.75,2.06)	**1.81 (1.15,2.84)**	**1.72 (1.05,2.81)**	0.67 (0.41,1.08)	**0.57 (0.33,0.98)**
Q4	**2.89 (1.92,4.34)**	**2.98 (1.54,5.77)**	**2.28 (1.04,5.01)**	0.91 (0.23,3.71)	**1.64** **(1.07,2.52)**	0.90 (0.43,1.87)	**3.84 (2.69,5.47)**	**3.03 (1.60,5.75)**	**2.78 (1.66,4.65)**	**2.28 (1.19,4.37)**	**0.62 (0.39,0.99)**	**0.40 (0.19,0.84)**
P for trend	**0.01**	0.13	**0.05**	0.15	0.91	0.23	**0.002**	**0.04**	**0.04**	0.33	0.47	0.62

CI, confidence interval; OR, odds ratio; EO, ethylene oxide; MetS, metabolic syndrome; EWC, elevated waist circumference; RHDL-C, reduced high-density lipoprotein cholesterol; ETGs, elevated total triglycerides.

Model 1: adjusted for age, sex, race, BMI;

Model 2: adjusted for variables in Model 2 plus marital status, educational level, drinking status, smoking status, hypertension, diabetes, PIR.

Quartiles for ethylene oxide (<22.62, 22.62-33.515, 33.515-148, >148) pmol/g Hb.

The bold values represent that the p-value associated with the respective Odds Ratio (OR) is less than 0.05.

### 3.3 Dose-response analysis between HbEO and MetS

**Figure 2:**
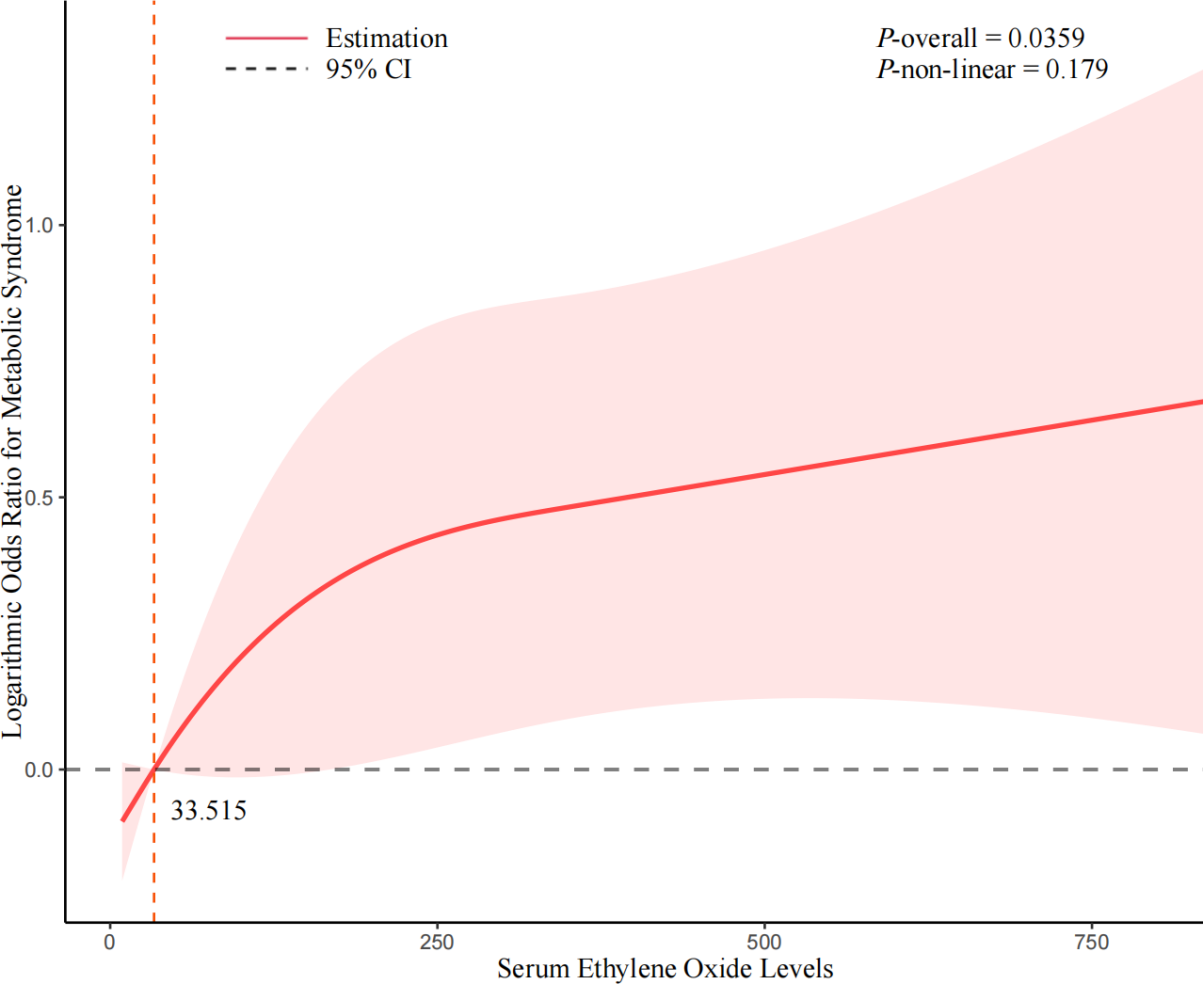
Non-linear association between serum ethylene oxide and the risk of Metabolic Syndrome. Cubic spline models adjusted for age (years), sex, BMI (<30 or ≥ 30kg/m2), race/ethnicity (Mexican American, Non-Hispanic Black, Non-Hispanic White, Other Race), educational level (9-11th grade or below, high school grad/GED or equivalent, college graduate or above, some college or AA degree), marital status (married/living with partner, widowed/divorced/separated, never married), smoking status (former, now, or never), drinking status (former, now, or never), PIR, diabetes (no, pre-diabetes, or diabetes mellitus), hypertension (yes or no). Knots = 3. Abbreviations: BMI, body mass index; CI, confidence interval.

The dose-response association between HbEO level and MetS risk indicated that increasing levels of serum HbEO were associated with a higher risk of MetS (**Figure 2**). However, the *p*-value for non-linearity was more significant than 0.05, suggesting a possible linear association between serum HbEO and MetS risk (*P*-overall=0.0359, *P*-non-linear=0.179). For the female population, there was a non-linear and L-shape association between HbEO level and MetS risk (*P*-overall<0.001, *P*-non-linear=0.0024) (refer to **Supplementary Figure 2**). Regarding the population without obesity, a significantly non-linear and inverted U-shape association was found between HbEO and MetS risk (*P*-overall=0.0107, *P*-non-linear=0.0055) (refer to **Supplementary Figure 3**). For the population aged 50 and older, a significantly non-linear and inverted U-shape association was found between HbEO and MetS risk (*P*-overall<0.001, *P*-non-linear=0.0157) (refer to **Supplementary Figure 4**).

### 3.4 Sensitive analysis

In sensitivity analysis (**Supplementary Table 1**) that used the IDF definition for MetS, the serum HbEO levels were positively associated with MetS risk in Q3 and Q4 levels (OR=1.71, 95% CI: 1.13~2.58; OR=2.60, 95% CI: 1.69~3.99, respectively) in model 1 with a significant *p*-value for trend (*P*=0.02). In model 2, serum HbEO levels were positively associated with MetS risk in Q4 level (OR=2.65, 95% CI: 1.35~5.19), with a non-significant *p*-value for trend (*P*=0.14). The analysis elucidated a positive correlation between logarithmic-transformed HbEO [Log (HbEO)] levels and the susceptibility to MetS (refer to **Supplementary Table 2**). In Model 1, the odds ratio (OR) denoting the association between Log (HbEO) levels and MetS risk was determined to be 1.31 (95% CI: 1.12~1.53), while in Model 2, it was 1.26 (95% CI: 1.03~1.55). The positive association persisted after adjusting for the Healthy Eating Index-2015 score within the model (refer to **Supplementary Table 3**). In Model 1, a discernible elevation in the risk of MetS was observed concomitant with EO levels. For instance, relative to the reference group, the OR for MetS was 2.29 (95% CI: 1.25~4.20) in the Q2 level of EO levels, 1.96 (95% CI: 1.23~3.41) in the Q3 level, and 2.77 (95% CI: 1.65~4.66) in Q4 level. Similarly, in Model 2, the risk of MetS exhibited a progressive augmentation with elevated EO levels (OR=2.17, 95% CI: 1.05~4.49) in Q2 level; OR=2.09, 95% CI: 1.13~3.87) in Q3 level; OR=2.23, 95% CI: 1.10~4.53) in Q4 level. These findings underscore a consistent and statistically significant positive association between EO exposure and the predisposition to MetS, even after adjustment for plausible confounding variables such as the Healthy Eating Index-2015 score.

### 3.5 Subgroup analysis

**Figure 3:**
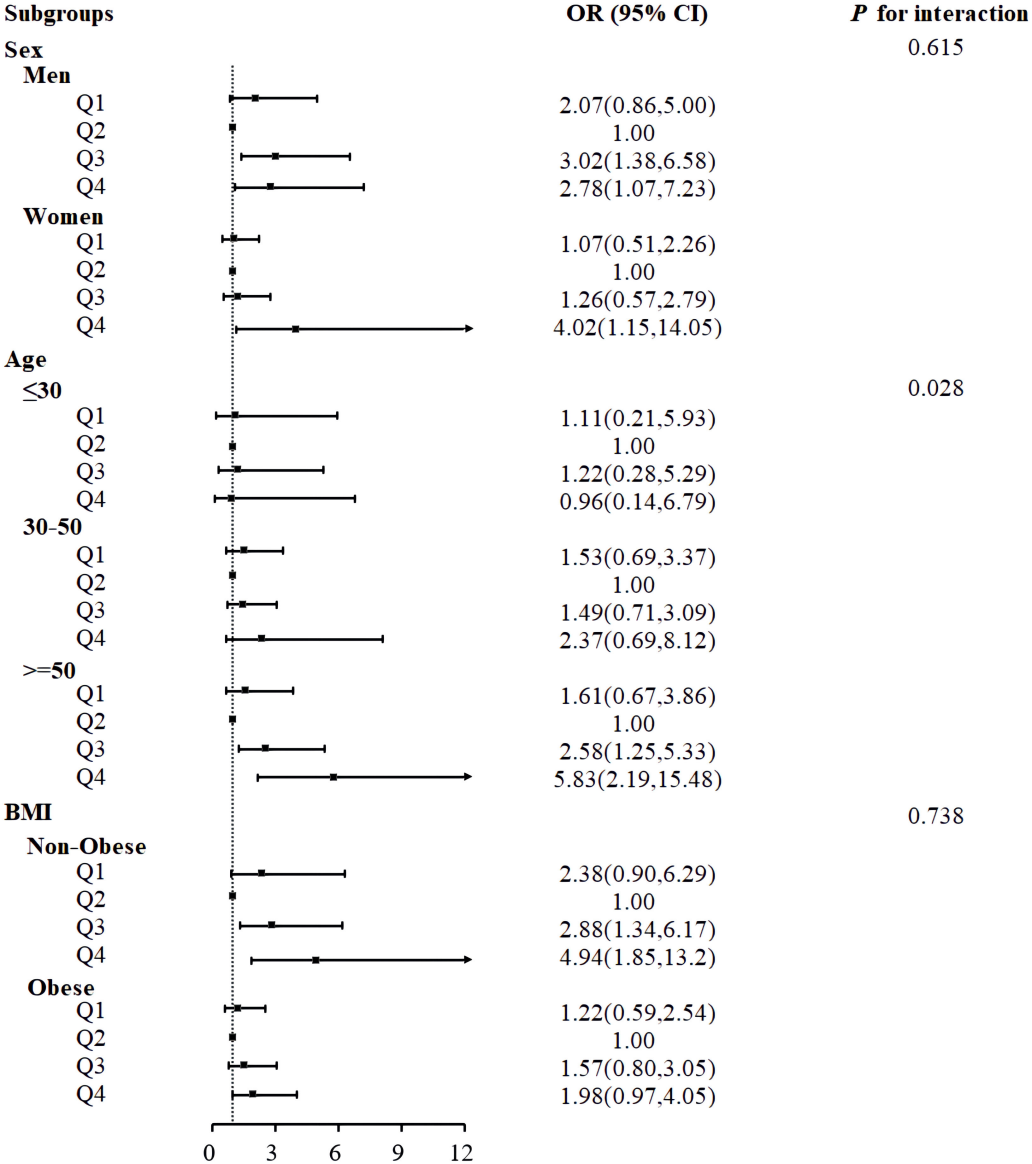
Associations between serum ethylene oxide and metabolic syndrome in subgroups. Models were adjusted for age (years), sex, BMI (<30 or ≥ 30kg/m^2^), race/ethnicity (Mexican American, Non-Hispanic Black, Non-Hispanic White, Other Race), educational level (9-11th grade or below, high school grad/GED or equivalent, college graduate or above, some college or AA degree), marital status (married/living with partner, widowed/divorced/separated, never married), smoking status (former, now, or never), drinking status (former, now, or never), PIR, diabetes (no, pre-diabetes, or diabetes mellitus), hypertension (yes or no).

Subgroup analysis was conducted to investigate potential sex, age, and BMI interactions with the relationship between serum HbEO and the risk of MetS (**Figure 3**). Notably, the subgroup analysis of age showed a significant difference between age groups (*P* for interaction=0.028), suggesting an interaction between serum HbEO and MetS about age. The serum HbEO level in males in Q3 and Q4 was significantly associated with an increased risk of MetS (OR=3.02, 95%CI: 1.38~6.58; OR=2.78, 95%CI: 1.07~7.23, respectively). This relationship was also observed in females in Q4 (OR=4.02, 95%CI: 1.15~14). There was a significant association between an increased risk of MetS and serum HbEO level in individuals aged 50 and over in Q3 and Q4 (OR=2.58, 95%CI: 1.25~5.33; OR=5.83, 95%CI: 2.19~15.48, respectively). Furthermore, the level of serum HbEO in individuals without obesity in Q3 and Q4 was also significantly associated with an increased risk of MetS (OR=2.88, 95%CI: 1.34~6.17; OR=4.94, 95%CI: 1.85~13.2, respectively). Subgroup analysis for hypertension and diabetes suggested hypertension and diabetes had no impact on the prevalence of MetS (*P*=0.411, *P*=0.993, respectively) (**Supplementary Figure 5**).

## 4 Discussion

This study demonstrated that HbEO is a risk factor for MetS and its components including elevated waist circumference, reduced high-density lipoprotein cholesterol and elevated total triglycerides. This finding remained consistent across subgroup analyses and sensitivity analyses. Grouped RCS curves revealed a notable increase in the risk of metabolic syndrome among women, non-obese individuals, and those aged over 50 years when exposed to EO.

Recent data suggest that MetS is the majority of the population’s attributable risk of premature death from cardiovascular disease (45). Although MetS appears more common in genetically predisposed people, acquired underlying risk factors—overweight or obesity and elevated waist circumference, insulin resistance, dyslipidemia, glucose intolerance, hypertension, physical inactivity, and atherosclerotic diet—often cause clinical manifestations (46). Environmental contamination also contribute to MetS development (25, 47). EO, as the reactive epoxide, mainly comes from the sterilization of chemical plants, commercial sterilization operations, and medical facilities (48, 49). Exposure to EO may lead to a range of adverse health effects, including angina, heart attack, total cardiovascular disease, dyslipidemia, and its genotoxicity and mutagenic abilities have been widely reported in several experimental studies (21, 22, 24, 27, 49, 50). The research of Zhu, Huang and Cheang suggests that the relationship between EO and lipid abnormalities, chronic obstructive pulmonary disease, and asthma is mediated by the inflammatory response caused by EO (22, 27, 28). Studies have found that exposure to EO can cause inflammation in rodent organs and promote the occurrence of pulmonary fibrosis in rodents (51, 52). Long-term chronic exposure to EO leads to a decrease in glutathione reductase activity and an increase in hepatic lipid peroxide associated with oxidative stress *in vivo*, which is thought to be an essential pathogenic mechanism involved in lipid metabolism (53–56). In the pathogenesis of MetS, inflammation related to obesity and overweight plays a significant role, contributing substantially to associated pathological outcomes (57). There is reason to believe that the mechanism of the increased risk of MetS due to elevated HbEO levels may be the pro-inflammatory effects of EO.

Notably, subgroup analysis found no sex disparity in MetS prevalence, which is in line with the findings of previous researches (58, 59). However, the L-shaped relationship between EO levels and the risk of MetS in the female group was also found in grouped RCS analysis, which is in line with what Assmann (60) discovered. Therefore, there is a certain contradiction in our results. With aging, there is a decline in sex hormones, leading to hormonal imbalance, resulting in an increase in testosterone levels and a decrease in estrogen levels in females (61). Estrogen acts at the cellular and organ levels mediated by α and β estrogen receptors, regulating feeding behavior, glucose utilization, insulin production, and visceral fat deposition (62). In the majority of women, post-menopause is not only characterized by redistribution of body weight but also by weight gain. Obesity and weight gain largely contribute to the increased prevalence of MetS after menopause (63). Central obesity also causes endocrine disruption through various mechanisms, including increased sensitivity of the hypothalamic-pituitary axis, increased cortisol, decreased gender-specific steroids, and increased adrenal androgens in women (61). However, the complex interplay among various biological and sex hormone-related factors in the underlying pathophysiology of MetS suggests that hormones do not solely drive gender-related disparities (64). While notable gender differences do exist, with females appearing to have a higher risk of MetS and males exhibiting a higher cardiovascular risk, these differences are not solely attributable to hormonal influences. In addition to hormones and genetic factors, factors such as binge eating, reduced physical activity, cultural expectations, educational attainment, and socioeconomic status contribute to gender and geographical disparities (65). In summary, future research should delve into the contribution of HbEO to MetS in different gender contexts.

MetS is typically closely associated with obesity, which not only constitutes a component of MetS but also serves as an independent risk factor contributing to its development. However, the grouped RCS curve reveals that the risk of MetS shows a rapid increase followed by a gradual decrease with increasing levels of EO in the non-obese group, a pattern not observed in the obese group. Studies exploring the relationship between obesity and HbEO have indicated a negative correlation (24). Therefore, the effect of obesity on HbEO may counterbalance the effect of EO on metabolic syndrome, offering a partial explanation for this phenomenon. On the other hand, obesity plays a significant role in exerting adverse effects on major cardiovascular risk factors (including hypertension, dyslipidemia, and diabetes), being a principal component of metabolic syndrome, and may act as an independent risk factor for atherosclerosis and cardiovascular events (66). However, some reports indicate that overweight and obese patients with coronary heart disease have lower overall mortality and cardiovascular mortality risks compared to those with underweight and normal weight (67). This is the obesity paradox, and HbEO is likely to participate in this mechanism by affecting the body’s inflammatory response (66). However, more and deeper mechanisms need to be explored, and more robust evidence can be provided. Furthermore, any acute disruption of a physiological regulatory system tends to elicit a response aimed at restoring balance. When being stimuli, changes in one system and homeostasis affect another system (57). Therefore, when HbEO levels are low, the immune system may be the first to respond, releasing inflammatory factors. When the concentration of HbEO reaches a certain threshold, other systems in the body also react to HbEO or when the concentration of HbEO reaches a threshold, the immune system also happens to reach homeostatic equilibrium. This is evidenced by the fact that as HbEO concentrations continue to rise, the risk of metabolic syndrome begins to decrease. This suggests that non-obese individuals may be more susceptible to the effects of epoxyethane exposure, thereby bearing a higher risk.

The average age of individuals diagnosed with MetS was observed to be higher than that of those without MetS, suggesting an age-related impact on the susceptibility to MetS in relation to EO exposure. This finding aligns with the outcomes of several previous investigations (68–71). With advancing age, the incidence of central obesity, hypertension, diabetes, dyslipidemia, and hormonal imbalances such as declining sex hormone levels may collectively contribute to the escalation of MetS prevalence (72). Notably, among individuals aged over 50, a distinctive L-shaped association between EO levels and MetS risk was discerned, a pattern not evident among those under 30 or between the ages of 30 and 50. Considering the age-related decline in physical performance and the accumulation of HbEO in the body alongside diminished HbEO metabolism, a convergence of factors partially elucidates the heightened vulnerability to MetS among middle-aged and elderly individuals exposed to EO.

This research investigates the relationship between exposure to EO and the risk of MetS, and between exposure to HbEO and the risk of the components of Mets, adding new evidence to the exploration of the pathogenesis of MetS, enriches the etiology of MetS, and provides a new direction for the treatment of it. Secondly, the data used to analyze was from a nationally representative series of surveys, so it was advantageous to generalize the findings gained from this study. At the same time, the limitations also should be mentioned. Firstly, this is a retrospective study, so that the causal relationship cannot be detected. Secondly, the questionnaire contains recall questions, which would cause bias. Finally, the mechanism underlying how EO exposure increases the risk of MetS has not been extensively explored in the current article. Therefore, additional studies are warranted to elucidate this mechanism and provide robust evidence. By conducting more relevant studies, we can strengthen the evidence base and gain deeper insights into the relationship between EO exposure and MetS risk.

## 5 Conclusion

This study demonstrates that HbEO is a risk factor for MetS and its components. As the levels of HbEO increase, the risk of developing MetS continues to rise. The risk of MetS associated with exposure to HbEO varies depending on gender, age, and BMI.

## Data availability statement

Publicly available datasets were analyzed in this study. This data can be found here: https://www.cdc.gov/nchs/nhanes/index.htm.

## Ethics statement

The studies involving humans were approved by National Center for Health Statistics Ethics Review Board. The studies were conducted in accordance with the local legislation and institutional requirements. The participants provided their written informed consent to participate in this study.

## Author contributions

CZ: Writing – original draft, Writing – review & editing. LJ: Data curation, Writing – review & editing, Investigation. SW: Writing – review & editing, Data curation, Methodology. RZ: Data curation, Writing – review & editing. YY: Data curation, Writing – review & editing. LY: Conceptualization, Supervision, Writing – review & editing.
