# Corrigendum: Positive association between blood ethylene oxide levels and metabolic syndrome: NHANES 2013-2020

**DOI:** 10.3389/fendo.2024.1429501

**Published:** 2024-05-29

**Authors:** Chunqi Zhou, Senlin Wang, Lingling Ju, Ruimin Zhang, Yunning Yang, Yanjun Liu

**Affiliations:** ^1^ College of Medicine, Southwest Jiaotong University, Chengdu, Sichuan, China; ^2^ Department of General Surgery, The Third People’s Hospital of Chengdu, Affiliated Hospital of Southwest Jiaotong University, College of Medicine, Southwest Jiaotong University, Chengdu, Sichuan, China

**Keywords:** metabolic syndrome, ethylene oxide, inflammation, epidemiology, NHANES

In the published article, there was an error in the second affiliation. Instead of “Department of General Surgery, Affiliated Hospital of Southwest Jiaotong University, College of Medicine, Southwest Jiaotong University, Chengdu, Sichuan, China”, it should be “Department of General Surgery, The Third People’s Hospital of Chengdu, Affiliated Hospital of Southwest Jiaotong University, College of Medicine, Southwest Jiaotong University, Chengdu, Sichuan, China”.

In the published article, there was an error in Yanjun Liu’s affiliation. Yanjun Liu was not affiliated with “College of Medicine, Southwest Jiaotong University, Chengdu, Sichuan, China” and this has been removed from his affiliations.

The authors apologize for these errors and state that they do not change the scientific conclusions of the article in any way. The original article has been updated.

