# Supplementary material for: Positive association between blood ethylene oxide levels and metabolic syndrome: NHANES 2013-2020
Source: Front Endocrinol (Lausanne). 2024 Apr 18;15:1365658. doi: 10.3389/fendo.2024.1365658 (PMC11063307; doi:10.3389/fendo.2024.1365658)
Supplement: Supplementary file 1 [file Table_1.docx]

**Supplementary Table 1. Multivariate weighted sensitivity logistics model analysis reveals the association between EO levels and** **the risk of** **Metabolic Syndrome.**

| EO Group | Case/  Participants^*^ | Model 1  OR (95% CI) | Model 2  OR (95% CI) |
| --- | --- | --- | --- |
| Q1 | 166/459 | 1.00 | 1.00 |
| Q2 | 167/448 | 1.54 [0.97,2.46] | 1.45 [0.85,2.48] |
| Q3 | 156/456 | **1.71 [1.13,2.58]** | 1.62 [0.95,2.75] |
| Q4 | 190/479 | **2.60 [1.69,3.99]** | **2.65 [1.35,5.19]** |

**Abbreviations:** CI, confidence interval; OR, odds ratio. Metabolism; EO, ethylene oxide.

Model 1: adjusted for age, sex, race, BMI;

Model 2: adjusted for variables in Model 2 plus marital status, educational level, drinking status, smoking status, hypertension, diabetes, PIR.

Quartiles for ethylene oxide (<22.62, 22.62-33.515, 33.515-148, >148) pmol/g Hb.

**Supplementary Table 2. Multivariate weighted sensitivity logistics model analysis reveals the association between Log (HbEO) levels and the risk of Metabolic Syndrome.**

| Characteristic | Case/  Participants^*^ | Model 1  OR (95% CI) | Model 2  OR (95% CI) |
| --- | --- | --- | --- |
| HbEO | 608/1842 | **1.31 [1.12,1.53]** | **1.26 [1.03,1.55]** |

**Abbreviations:** CI, confidence interval; OR, odds ratio. Metabolism; EO, ethylene oxide.

Model 1: adjusted for age, sex, race, BMI;

Model 2: adjusted for variables in Model 2 plus marital status, educational level, drinking status, smoking status, hypertension, diabetes, PIR.

Quartiles for ethylene oxide (<22.62, 22.62-33.515, 33.515-148, >148) pmol/g Hb.

**Supplementary Table 3. Multivariate weighted sensitivity logistics model analysis reveals the association between EO levels and the risk of Metabolic Syndrome.**

| EO Group | Case/  Participants^*^ | Model 1  OR (95% CI) | Model 2  OR (95% CI) |
| --- | --- | --- | --- |
| Q1 | 54/121 | 1.00 | 1.00 |
| Q2 | 91/182 | **2.29 [1.25,4.20]** | **2.17 [1.05,4.49]** |
| Q3 | 98/218 | **1.96 [1.12,3.41]** | **2.09 [1.13,3.87]** |
| Q4 | 124/223 | **2.77 [1.65,4.66]** | **2.23 [1.10,4.53]** |

**Abbreviations:** CI, confidence interval; OR, odds ratio. Metabolism; EO, ethylene oxide.

Model 1: adjusted for age, sex, race, BMI;

Model 2: adjusted for variables in Model 2 plus marital status, educational level, drinking status, smoking status, hypertension, diabetes, PIR, and Healthy Eating Index-2015 score.

Quartiles for ethylene oxide (<22.62, 22.62-33.515, 33.515-148, >148) pmol/g Hb.
