# Supplementary material for: Positive association between blood ethylene oxide levels and metabolic syndrome: NHANES 2013-2020
Source: Front Endocrinol (Lausanne). 2024 Apr 18;15:1365658. doi: 10.3389/fendo.2024.1365658 (PMC11063307; doi:10.3389/fendo.2024.1365658)
Supplement: Supplementary file 2 [file DataSheet_1.pdf]

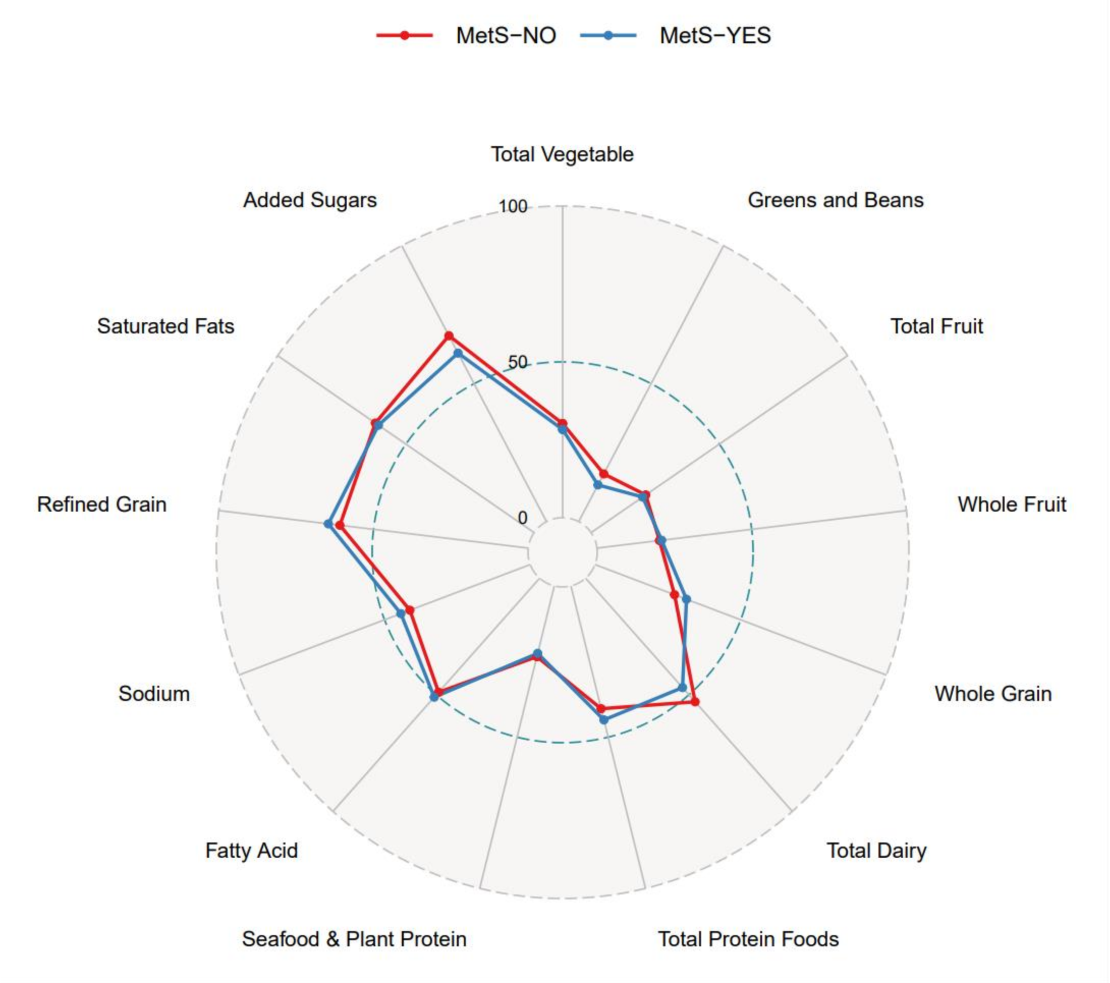

Supplementary Figure 1. The component scores of the Healthy Eating Index-2015 are represented as a percentage of the total possible component score for adults, where metabolic syndrome is defined according to the NCEP ATP III-2005 criteria. The Healthy Eating Index-2015 is a weighted score, with MetS-YES (those diagnosed with metabolic syndrome, n = 367) and MetS-NO (those diagnosed without metabolic syndrome, n = 744). The population ratio method was used to calculate the mean intake of each group. Dairy, total protein foods, and seafood and plant proteins include alternative dairy and protein products such as soy. The numbers represent the percentage consumed from 0 to 100.

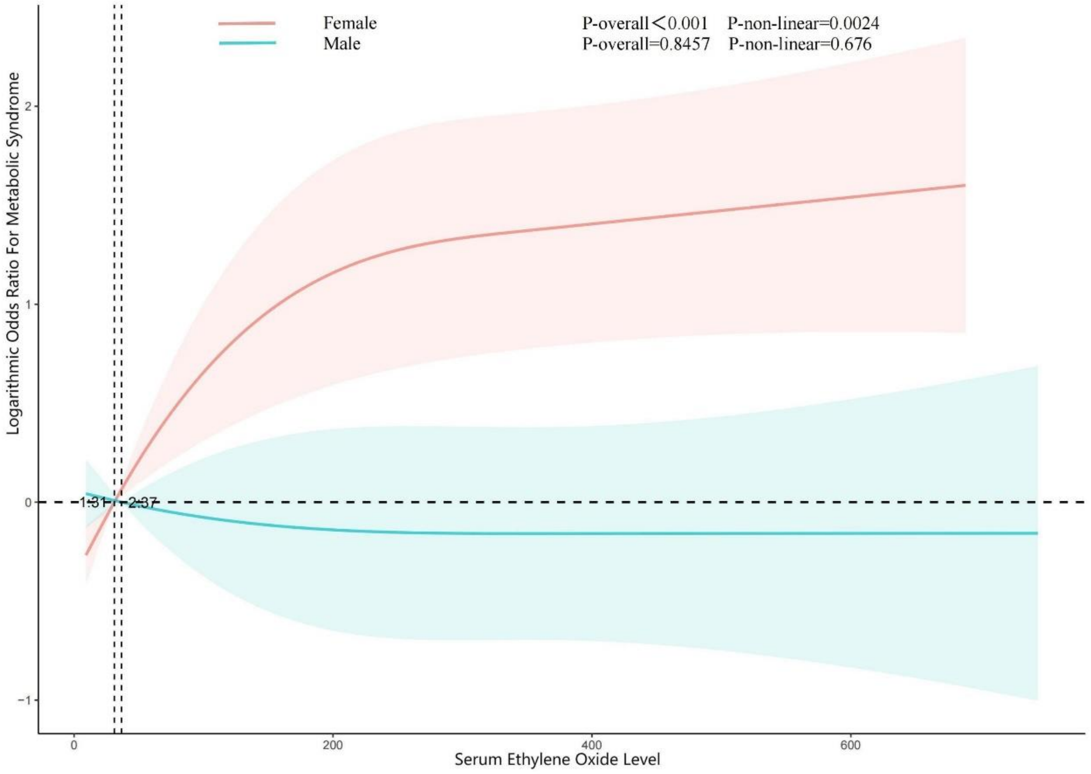

Supplementary Figure 2. Non-linear association between serum ethylene oxide and the risk of Metabolic Syndrome in male and female population. Cubic spline models adjusted for age (years), BMI (<30 or ≥ 30kg/m^2), race/ethnicity (Mexican American, Non-Hispanic Black, Non-Hispanic White, Other Race), educational level (9-11th grade or below, high school grad/GED or equivalent, college graduate or above, some college or AA degree), marital status (married/living with partner, widowed/divorced/separated, never married), smoking status (former, now, or never), drinking status (former, now, or never), PIR, diabetes (no, pre-diabetes, or diabetes mellitus), hypertension (yes or no). Knots = 3. Abbreviations: BMI, body mass index; CI, confidence interval.

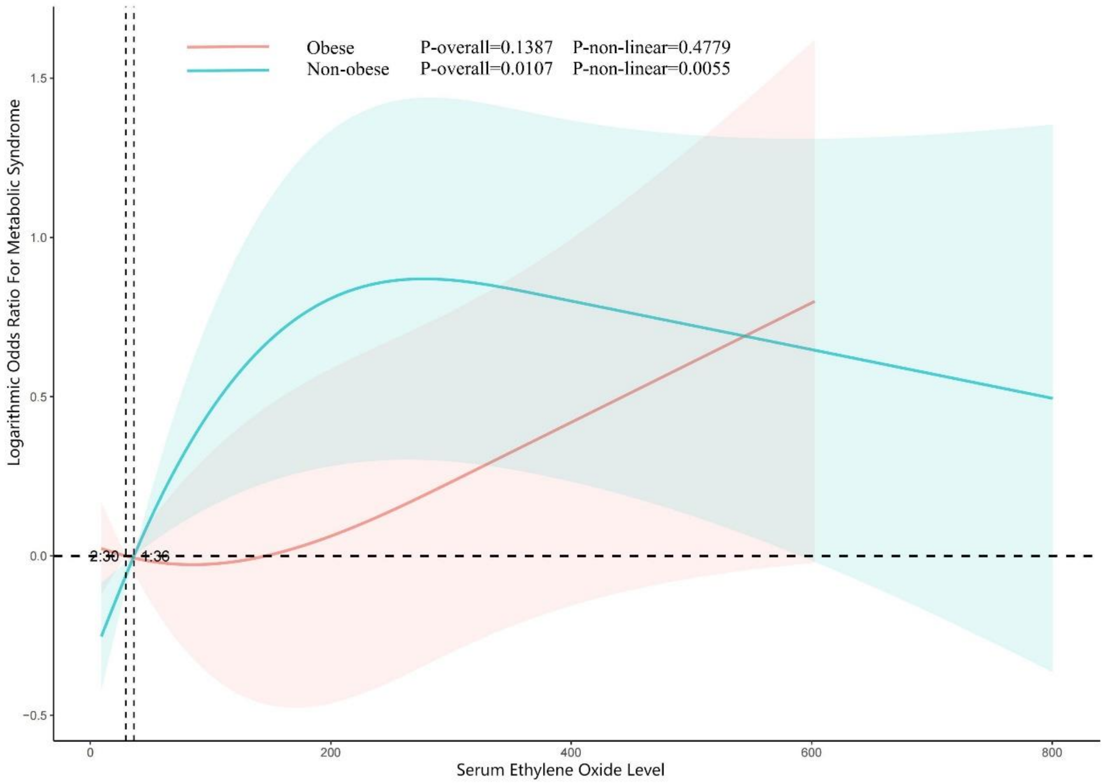

Supplementary Figure 3. Non-linear association between serum ethylene oxide and the risk of Metabolic Syndrome in obese and non-obese population. Cubic spline models adjusted for age (years), sex, race/ethnicity (Mexican American, Non-Hispanic Black, Non-Hispanic White, Other Race), educational level (9-11th grade or below, high school grad/GED or equivalent, college graduate or above, some college or AA degree), marital status (married/living with partner, widowed/divorced/separated, never married), smoking status (former, now, or never), drinking status (former, now, or never), PIR, diabetes (no, pre-diabetes, or diabetes mellitus), hypertension (yes or no). Knots = 3. Abbreviations: BMI, body mass index; CI, confidence interval.

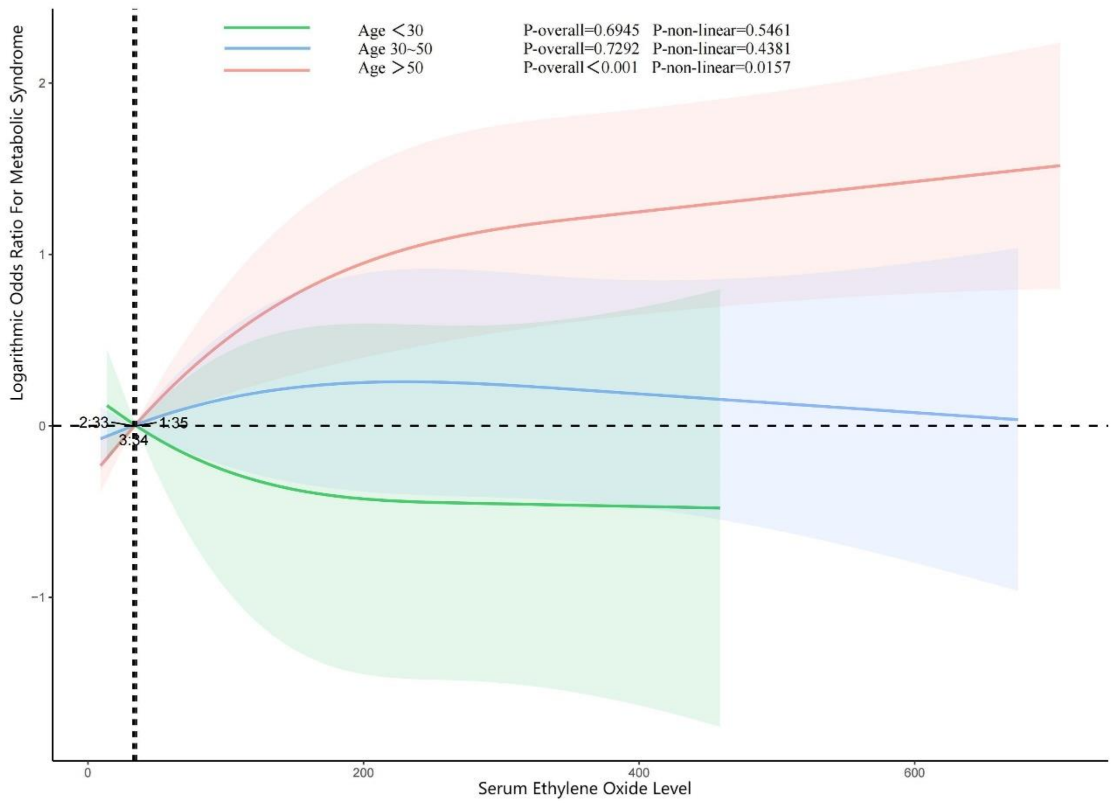

Supplementary Figure 4. Non-linear association between serum ethylene oxide and the risk of Metabolic Syndrome in different age population. Cubic spline models adjusted for sex, BMI (<30 or ≥ 30kg/m^2), race/ethnicity (Mexican American, Non-Hispanic Black, Non-Hispanic White, Other Race), educational level (9-11th grade or below, high school grad/GED or equivalent, college graduate or above, some college or AA degree), marital status (married/living with partner, widowed/divorced/separated, never married), smoking status (former, now, or never), drinking status (former, now, or never), PIR, diabetes (no, pre-diabetes, or diabetes mellitus), hypertension (yes or no). Knots = 3. Abbreviations: BMI, body mass index; CI, confidence interval.

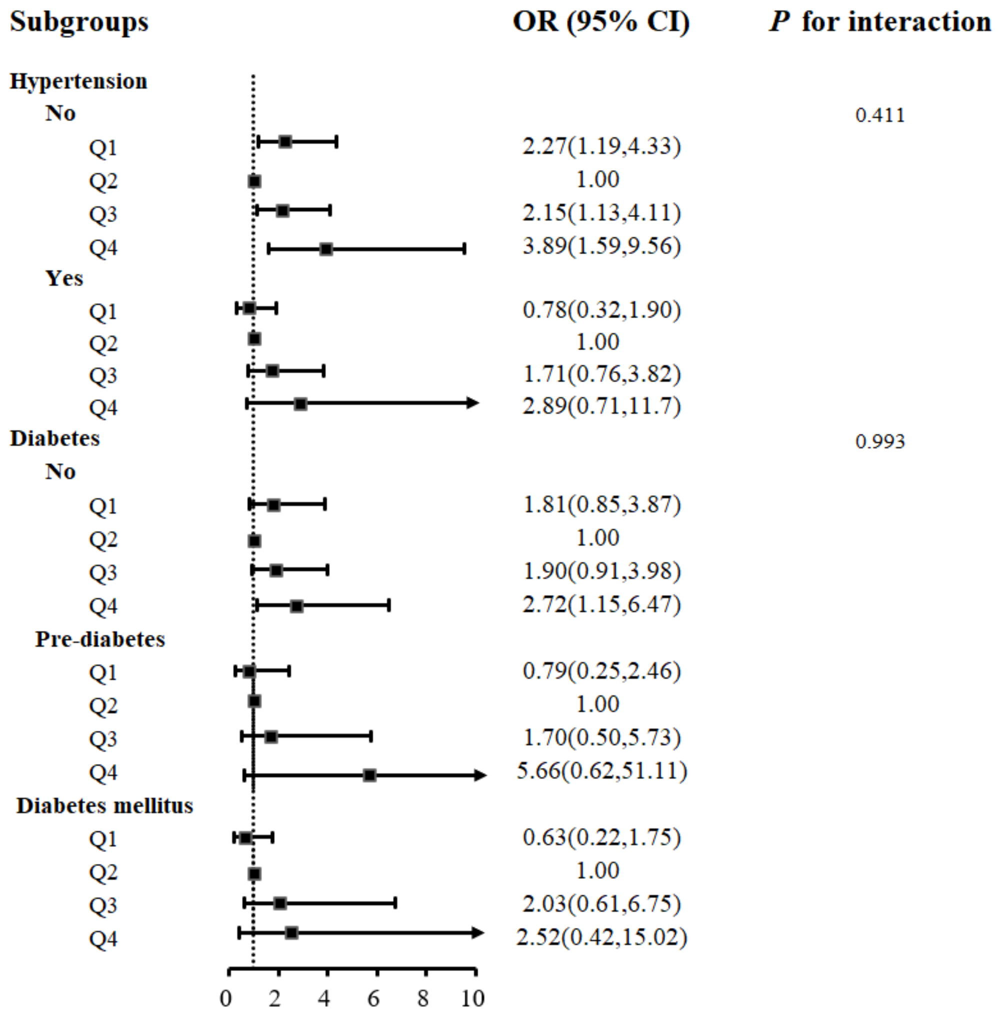

Supplementary Figure 5. Associations between serum ethylene oxide and metabolic syndrome in subgroups. Models were adjusted for age (years), sex, BMI (<30 or ≥ 30kg/m^2), race/ethnicity (Mexican American, Non-Hispanic Black, Non-Hispanic White, Other Race), educational level (9-11th grade or below, high school grad/GED or equivalent, college graduate or above, some college or AA degree), marital status (married/living with partner, widowed/divorced/separated, never married), smoking status (former, now, or never), drinking status (former, now, or never), PIR, diabetes (no, pre-diabetes, or diabetes mellitus), hypertension (yes or no). Knots = 3. Abbreviations: BMI, body mass index; CI, confidence interval; OR, odds ratio.
